# Tobacco and alcohol use are the risk factors responsible for the greatest burden of head and neck cancers: a study from the Global Burden of Disease Study 2019

**DOI:** 10.1080/07853890.2025.2500693

**Published:** 2025-05-03

**Authors:** Yue Yuan, Jing-wen Huang, Jia-lin Cao, Jian-hui Wu, Lu-ling Wang, Hui Gan, Jian-hui Xu, Fei Ye

**Affiliations:** ^a^ Department of Otolaryngology Head and Neck Surgery, Zhongshan City People’s Hospital, Zhongshan City, Guangdong Province, China; ^b^ Faculty of Chinese Medicine, Macao University of Science and Technology, Macao, China; ^c^ Department of Dermatology, Zhongnan Hospital of Wuhan University, Wuhan, China; ^d^ Department of Otolaryngology Head and Neck Surgery, Huangpu Hospital, Zhongshan City, Guangdong Province, China

**Keywords:** Global burden of disease, head and neck cancer, risk factors, smoking, alcohol use

## Abstract

**Background:**

The risk factors associated with cancers of the larynx, nasopharynx, lips, and oral cavity, as well as other pharyngeal cancers, share many similarities. To better understand how these risk factors manifest differently across various head and neck tumor types, we utilized data from the GBD database to conduct an in-depth analysis.

**Methods:**

Our study employed the 2019 GBD dataset to scrutinize trends in incidence, mortality, and DALYs related to these cancers. This analysis covered the period from 1990 to 2019 and was stratified by sex, age, geographical region, and the socio-demographic index.

**Findings:**

In 2019, lip and oral cavity cancers were found to have the highest incidence rates, with notably higher ASIRs observed in males compared to females. Interestingly, the ASIRs for laryngeal cancer showed a decreasing trend over the studied time frame from 1990 to 2019. Our findings revealed that smoking posed a significantly greater risk for laryngeal and lip and oral cavity cancers, whereas alcohol consumption was more strongly linked to NPC. Central Europe exhibited the ASDR for laryngeal cancer. For lip and oral cavity cancer, the impact of tobacco chewing on female ASDR was most pronounced in South Asia. In contrast, nasopharyngeal cancer had its highest ASDR in Asia.

**Conclusion:**

Our investigation underscores that smoking and alcohol consumption are leading risk factors for cancers of the head and neck, although their effects vary depending on the specific type of cancer, the sex of the patient, age group, and regional demographics. While occupational exposure to carcinogenic substances does not appear to be a predominant factor, it remains an important consideration that should not be overlooked in the comprehensive assessment of risk for these malignancies.

## Introduction

Head and neck tumors are a group of neoplasms that develop in the anatomical region of the head and neck. According to GLOBOCAN estimates of cancer incidence in 2020, head and neck cancers accounted for more than 4.8% of all cancers in the world [1]. It is estimated that hundreds of thousands of people die from head and neck cancers each year, posing a serious threat to global health [2]. Epidemiological studies have shown that the incidence of head and neck cancer is related to many factors. Smoking and alcohol abuse are one of the most important risk factors for head and neck cancer, and long-term and heavy smoking and alcohol abuse will increase the risk of head and neck cancer [3]. In addition, the occupational exposure of carcinogens is also closely related to the incidence of head and neck tumors in the mouth, throat and other parts of the body [4,5].

The Global Burden of Disease (GBD) database is a huge database established by the cooperation of multiple institutions and organizations around the world, which is used to assess the global burden of diseases, injuries and risk factors on human health [6]. The GBD database aims to provide detailed data on the burden of disease, mortality, disability and health-related risk factors to assist governments, health institutions and researchers in research, policy development and intervention design for a variety of diseases and health problems [6,7]. The GBD database contains a large amount of global data on head and neck malignancies, and uses standardized methods and models to integrate and analyze these data. In the GBD database, head and neck cancers are mainly divided into larynx cancer(D02.000), nasopharynx cancer(NPC)(D00.000 × 007), lip and oral cavity cancer(D00.000) and other pharynx cancer. The GBD database allows us to obtain, compare and analyze detailed information on various diseases, injuries, causes of death, disability, indicators of health risk factors and more.

In our research, we identified several common risk factors among larynx cancer, nasopharynx cancer, lip and oral cavity cancer, and other pharynx cancers. To better understand the specific variations in these risk factors across different types of head and neck cancers, we extracted data from the GBD database. We categorized this data by age, gender, and geographical region. Our primary goal was to investigate how these risk factors differ among various head and neck cancers, providing insights that can inform targeted prevention strategies.

## Methods

### Data sources

The Global Burden of Disease (GBD) database contains statistics on 369 diseases in 204 countries or regions. Data including the incidence, mortality, disability adjusted life years (DALYs) and the corresponding age-standardized rate (ASRs) (https://vizhub.healthdata.org/gbd-results/). We downloaded global information on sex, age, sociodemographic index (SDI) and potential risk factors associated with head and neck cancers, including incident numbers, deaths, ASRs and DALYs, to assess their impact on disease burden.

The GBD uses 100,983 data sources that cover a variety of types, including vital registration systems, verbal autopsy, censuses, household surveys, disease-specific registries, health service contact data, and more. These data sources provide detailed information on diseases and injuries to estimate incidence, mortality, years of disability (YLDs) and disability-adjusted life years (DALYs).

In the GBD database, head and neck cancers are mainly divided into larynx cancer (D02.000), nasopharynx cancer (NPC, D00.000 × 007), lip and oral cavity cancer (D00.000) and other pharynx cancer. Risk factors for these cancers include Tobacco, Alcohol use and Occupational carcinogens. Tobacco includes Smoking and Chewing tobacco, Occupational carcinogens include Occupational exposure to asbestos, Occupational exposure to sulfuric acid, and Occupational exposure to formaldehyde. Occupational exposure to asbestos and Occupational exposure to sulfuric acid were only associated with Larynx cancer. Occupational exposure to formaldehyde was only associated with NPC. Chewing tobacco was only associated with Lip and oral cavity cancer.

### Statistic analysis

The number of incidence, number of deaths, disability-adjusted life years (DALYs), and age-standardized rates (ASRs) were utilized for evaluating trends in head and neck tumour incidence and mortality. DALYs were calculated as the sum of years of life lost (YLLs) and years lived with disability (YLDs). Disability-adjusted life expectancy can be considered a measure of "healthy life" lost. ASR included age-standardized incidence rate (ASIR), age-standardized death rate (ASDR), and age-standardized DALY rate. The ASRs provided in the GBD are considered objective measures for quantifying trends in cancer incidence. Comparisons are made among populations with different age structures or over time within a particular population’s age distribution.

The epidemiological status of head and neck tumors was analyzed by cross-sectional comparison of various data (ASIR, ASDR, DALY, etc.). We present risk data for head and neck tumors from three perspectives (sex, age, and region) and analyze the differences among different head and neck tumors.

### Data visualization

All analyses were conducted using the open source software R, version 3.6.3. Data visualization utilized packages such as ggplot2, Rcolor Brewer, and cowplot. Image processing was carried out using GraphPad Prism software version 8.3.0.

## Results

### Rate and number of incidence, mortality and disability-adjusted life years (DALYs)

In 2019, lip and oral cavity cancers had the highest ASIR and ASDR among head and neck cancers, with the ASIR and ASDR of 4.52/100 000 (4.13 to 4.89) and 2.44/100 000 (2.22 to2.66), respectively. Compared with 1990, the ASIR of lip and oral cavity cancer increased slightly to 5.48% (–5.71 to 15.86), the mortality rate remained almost unchanged, and the DALYs decreased slightly to −1.42% (−14.20 to 11.21) (Table 1).

**Table 1.** Global incidence, prevalence, deaths, DALYs of head and neck cancer.

Measure#	Age (metric)	Year	Cause			
			Nasopharynx cancer	Lip and oral cavity cancer	Larynx cancer	Other pharynx cancer
Incidence	All ages (number)	% Change*	161.41 (124.43 to 204.45)	112.43 (89.58 to 133.69)	67.80 (55.27 to 82.54)	150.86 (120.36 to 176.50)
		2019	176501.78 (156046.19 to 199917.07)	373097.83 (340884.33 to 403865.82)	209148.87 (193875.74 to 224620.29)	166901.24 (152960.05 to 180358.71)
	Age-standardised (rate per 100,000)	% Change	37.10 (18.20 to 59.38)	5.48 (-5.71 to 15.86)	−17.92 (-23.96 to −10.82)	24.70 (9.74 to 37.50)
		2019	2.12 (1.87 to 2.40)	4.52 (4.13 to 4.89)	2.51 (2.32 to 2.69)	1.99 (1.83 to 2.15)
Prevalence	All ages (number)	% Change	299.18 (237.64 to 372.52)	119.83 (98.36 to 140.54)	79.77 (67.96 to 93.89)	240.02 (208.32 to 269.65)
		2019	971935.91 (845407.66 to 1119384.39)	1401931.91 (1286017.38 to 1519844.19)	1212790.06 (1127532.20 to 1304926.64)	264631.58 (243777.75 to 286390.50)
	Age-standardised (rate per 100,000)	% Change	115.32 (82.94 to 153.84)	12.00 (1.09 to 22.53)	−9.78 (-15.84 to −2.91)	70.45 (54.39 to 85.33)
		2019	11.65 (10.15 to 13.41)	16.82 (15.43 to 18.23)	14.52 (13.52 to 15.62)	3.14 (2.89 to 3.39)
Deaths	All ages (number)	% Change	33.95 (19.25 to 51.77)	106.35 (81.10 to 131.35)	41.04 (30.12 to 53.66)	121.93 (89.64 to 153.46)
		2019	71610.49 (65442.01 to 77624.61)	199397.55 (181651.48 to 218058.56)	123355.59 (114941.40 to 132798.43)	114206.72 (103153.62 to 126039.37)
	Age-standardised (rate per 100,000)	% Change	−31.34 (-38.86 to −22.45)	0 (-12.01 to 12.04)	−31.63 (-36.78 to −25.72)	9.54 (-6.19 to 24.98)
		2019	0.86 (0.79 to 0.93)	2.44 (2.22 to 2.66)	1.49 (1.39 to 1.61)	1.37 (1.24 to 1.51)
DALYs	All ages (number)	% Change	24.16 (9.73 to 41.06)	92.89 (67.78 to 117.64)	31.90 (21.06 to 44.35)	107.87 (77.21 to 137.45)
		2019	2335095.9 (2139753.36 to 2536657.38)	5506651.72 (5004325.43 to 6033424.29)	3262221.4 (3034634.21 to 3511354.39)	3234593.10 (2904981.32 to 3571833.38)
	Age-standardised (rate per 100,000)	% Change	−32.86 (-40.60 to −23.84)	−1.42 (-14.20 to 11.21)	−34.22 (-39.58 to −28.08)	5.67 (-9.84 to 20.81)
		2019	41.67 (38.08 to 45.35)	67.01 (62.93 to 71.57)	38.83 (36.13 to 41.78)	38.44 (34.51 to 42.44)

#Data in parentheses are 95% Uncertainty Intervals (95% UIs).

*% Change (1990–2019).

Among the head and neck cancers, the ASIR and ASDR of laryngeal cancer were second only to those of lip and oral cavity, which were 2.51/100 000 (2.32 to 2.69) and 1.49/100 000 (1.39 to 1.61), respectively. Notably, among the head and neck cancers, only the ASIR of laryngeal cancer decreased from −17.92% (−23.96 to −10.82) in 1990. The ASDR and DALY also showed a downward trend, which were −31.63% (-36.78 to −25.72) and −34.22% (−39.58 to −28.08), respectively.

The ASIR of NPC was 2.12/100 000 (1.87 to 2.40) in 2019, the number of new cases was 176501.78 (156046.19 to 199917.07), and the ASDR was 0.86/100 000 (0.79 to 0.93). The number of deaths was 71610.49 (77624.61 to 65442.01). Compared with 1990, the ASIR of NPC increased by 37.10% (18.20 to 59.38), while the ASDR decreased by −31.34% (−38.86 to −22.45). The age-standardized DALY rate decreased −32.86% (−40.60 to −23.84).

### Risk factors for head and neck cancer

**Figure 1.**
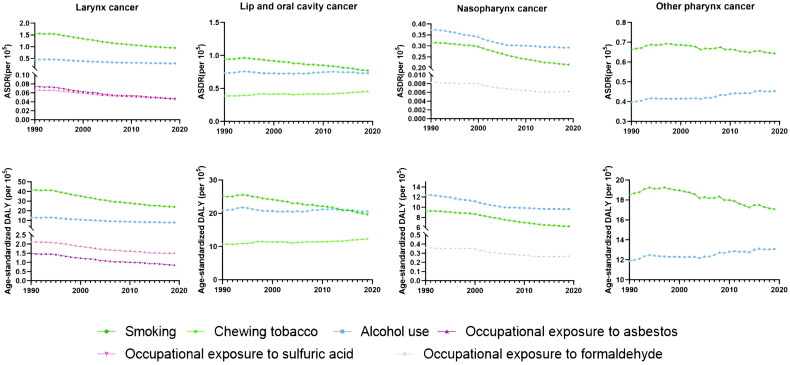
Age-standardized death rate (ASDR) and age-standardized DALY rate of head and neck cancer by risk factors worldwide from1990 to 2019. (A) larynx cancer; (B) lip and oral cavity cancer; (C) nasopharynx cancer; (D) other phraynx cancer.

In the GBD database, the main risk factors for larynx cancer were smoking, alcohol use, occupational exposure to sulfuric acid and asbestos (Table 2). Among them, smoking caused the highest number of laryngeal cancer deaths, 78269.08 (88323.69 to 67968.78) (Table S1), accounting for 63.45% of laryngeal cancer deaths in 2019, much higher than alcohol use (19.37%). Occupational exposure to asbestos and Occupational exposure to sulfuric acid accounted for only 2.98% and 3.27%. From 1990 to 2019, the ASDR of laryngeal cancer caused by smoking and alcohol use both showed a downward trend (Figure 1A), and the decline of smoking was more significant (39.58%).

**Table 2.** Global age-standardized death rates and disability-adjusted life years(DALYs) and percent changes for risk factors for head and neck cancer.

Measure#	Year	Risk factor	Cause(Age-standardised rate, per 100,000)			
			Nasopharynx cancer	Lip and oral cavity cancer	Larynx cancer	Other pharynx cancer
Deaths	% Change*	**Tobacco**	−31.30 (−41.08 to −19.36)	−7.46 (−20.23 to 5.55)	−39.58 (−44.76 to −33.95)	−3.07 (−18.14 to 10.44)
		Smoking	−31.30 (−41.08 to −19.36)	−18.61 (−29.92 to −7.27)	−39.58 (−44.76 to −33.95)	−3.07 (−18.14 to 10.44)
		Chewing tobacco		17.90 (−4.88 to 42.66)		
		**Alcohol use**	−21.52 (−33.89 to −7.51)	−0.32 (−12.75 to 11.98)	−37.05 (−42.83 to −30.85)	14.01 (−1.77 to 30.22)
		**Occupational carcinogens**	−24.77 (−36.33 to −11.06)		−33.37 (−40.24 to −26.20)	
		Occupational exposure to asbestos			−38.13 (−44.15 to −32.47)	
		Occupational exposure to sulfuric acid			−28.00 (−38.06 to −14.73)	
		Occupational exposure to formaldehyde	−24.77 (−36.33 to −11.06)			
	2019	**Tobacco**	0.21 (0.15 to 0.27)	1.12 (0.96 to 1.30)	0.94 (0.82 to 1.06)	0.64 (0.54 to 0.74)
		Smoking	0.21 (0.15 to 0.27)	0.77 (0.62 to 0.92)	0.94 (0.82 to 1.06)	0.64 (0.54 to 0.74)
		Chewing tobacco		0.45 (0.34 to 0.57)		
		**Alcohol use**	0.29 (0.22 to 0.35)	0.73 (0.58 to 0.88)	0.28 (0.17 to 0.39)	0.45 (0.34 to 0.56)
		**Occupational carcinogens**	0.006 (0.004 to 0.009)		0.09 (0.05 to 0.13)	
		Occupational exposure to asbestos			0.04 (0.02 to 0.07)	
		Occupational exposure to sulfuric acid			0.05 (0.02 to 0.08)	
		Occupational exposure to formaldehyde	0.006 (0.004 to 0.009)			
DALYs	% Change	**Tobacco**	−32.54 (−42.59 to −19.95)	−9.94 (−23.09 to 3.54)	−42.49 (−47.57 to −36.80)	−7.94 (−22.16 to 5.23)
		Smoking	−32.54 (−42.59 to −19.95)	−21.29 (−9.77 to −32.63)	−42.49 (−47.57 to −36.80)	−7.94 (−22.16 to 5.23)
		Chewing tobacco		14.67 (−8.24 to 39.70)		
		**Alcohol use**	−22.16 (−34.34 to −8.00)	−1.84 (−14.98 to 11.03)	−39.54 (−45.19 to −33.29)	10.32 (−4.92 to 26.84)
		**Occupational carcinogens**	−24.83 (−36.47 to −10.87)		−35.11 (−42.61 to −27.26)	
		Occupational exposure to asbestos			−42.43 (−48.20 to −36.56)	
		Occupational exposure to sulfuric acid			−30.04 (−40.19 to −16.76)	
		Occupational exposure to formaldehyde	−24.83 (−36.47 to −10.87)			
	2019	**Tobacco**	6.23 (4.43 to 8.09)	29.44 (24.78 to 34.48)	24.04 (20.85 to 27.27)	17.07 (14.20 to 19.92)
		Smoking	6.23 (4.43 to 8.09)	19.69 (15.61 to 23.93)	24.04 (20.85 to 27.27)	17.07 (14.20 to 19.92)
		Chewing tobacco		12.28 (9.13 to 15.73)		
		**Alcohol use**	9.64 (7.47 to 11.81)	20.57 (16.39 to 24.62)	7.77 (4.68 to 10.60)	13.09 (10.09 to 16.18)
		**Occupational carcinogens**	0.27 (0.18 to 0.38)		2.32 (1.37 to 3.64)	
		Occupational exposure to asbestos			0.86 (0.47 to 1.30)	
		Occupational exposure to sulfuric acid			1.48 (0.63 to 2.74)	
		Occupational exposure to formaldehyde	0.27 (0.18 to 0.38)			

#Data in parentheses are 95% Uncertainty Intervals (95% UIs).

*% Change(1990–2019).

The main risk factors for lip and oral cavity cancer are smoking, chewing tobacco and alcohol use. Smoking caused the highest number of deaths at 63,433.56 (76,386.46 to 51,216.43), accounting for 31.81% of deaths in 2019, followed by alcohol use at 30.27%. Chewing tobacco accounts for 18.71%. The ASDR caused by smoking showed a downward trend (18.61%) from 1990 to 2019. While chewing tobacco showed an upward trend (17.90%), the ASDR due to alcohol use was almost unchanged (−0.32%) (Figure 1B).

The associated risk factors for nasopharynx cancer are smoking, alcohol use, and occupational exposure to formaldehyde (Table 2). In 2019, there were 24,458.84 (29,947.73 to 18824.16) (Table S1) deaths related to nasopharyngeal cancer caused by alcohol use, accounting for the highest proportion of nasopharyngeal cancer deaths in that year, accounting for 34.15%. Followed by smoking accounted for 25.02%, and occupational exposure to formaldehyde accounted for only 0.72%.In the past 30 years, the ASDR caused by smoking showed the most significant downward trend (31.3%) (Figure 1C). The decrease of ASDR caused by alcohol use was 21.52%.

The common risk factors for Other pharynx cancers are smoking and alcohol use. Smoking caused the highest number of deaths at 53612.44 (45182.27 to 61925.36), accounting for 46.94% of the deaths in 2019, followed by drinking (33.16%). From 1990 to 2019, ASDR caused by smoking showed a downward trend (−3.07%). ASDR caused by drinking is on the rise (14.01%) (Figure 1D).

### Risk factors for age and sex

**Figure 2.**
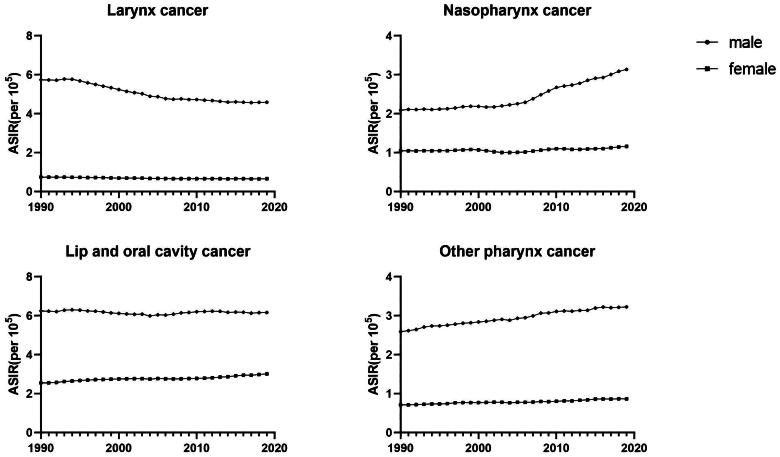
Age-standardized incidence rate(ASIR) of head and neck cancer in men and women worldwide from 1990 to 2019.

In 2019, the ASIR of all head and neck malignant tumors is greater in males than in females, and the ratio of male to female incidence of laryngeal cancer is the largest, which is about: 7.00:1, nasopharyngeal cancer is about: 2.71:1, and lip and oral cancer is about: 2.04:1.For NPC, ASIR increased significantly among men (1990-2019), while for laryngeal cancer, ASIR decreased significantly among men (Figure 2). When looking at the ASDR and age-standardized DALY rates from 1990 to 2019, it becomes evident that the disease burden of all head and neck cancers was greater in men compared to women (Figure S1-2).

**Figure 3.**
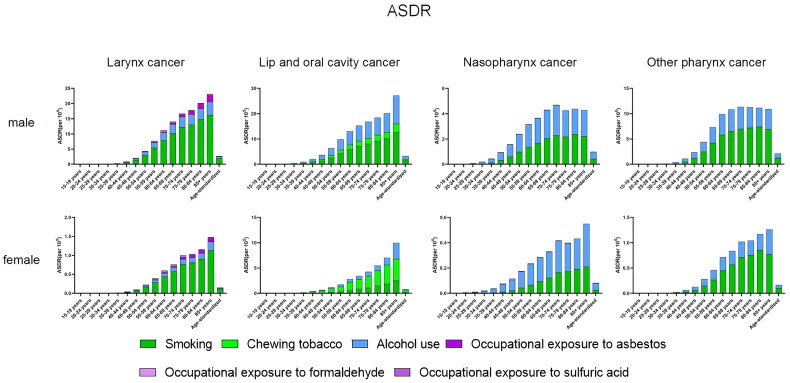
Absolute rate of death from head and neck cancer by age in men and women in 2019 and with age-standardised rate.ASDR:age-standardized death rate(per 100000).

**Figure 4.**
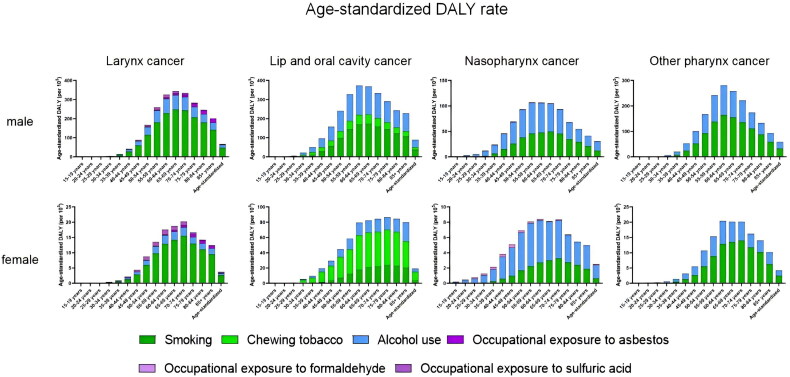
Absolute rate of DALY(per 100000) from head and neck cancer by age in men and women in 2019 and with age-standardised rate.

Regardless of male or female, the older the age group, the higher the ASDR of laryngeal cancer, and the ASDR of male laryngeal cancer is much higher than that of female. Smoking is the highest in all age groups (Figure 3). 70-74 years were the age group with the highest age-standardized DALY rate for laryngeal cancer (Figure 4).

Among men, the 70-74 age group has the highest ASDR of NPC, and the use of alcohol causes slightly more NPC related deaths than smoking. However, among women, the ASDR of NPC was higher in the older age group. The age group with the highest DALYs for NPC was 50-59 years.

Smoking was the main factor affecting the death of lip and oral cavity cancer in men, and it increased with age. In women, chewing tobacco was the leading cause of death due to lip and oral cancer.

### Risk factors for 5 SDI regions and 21 GBD regions in 2019

**Figure 5.**
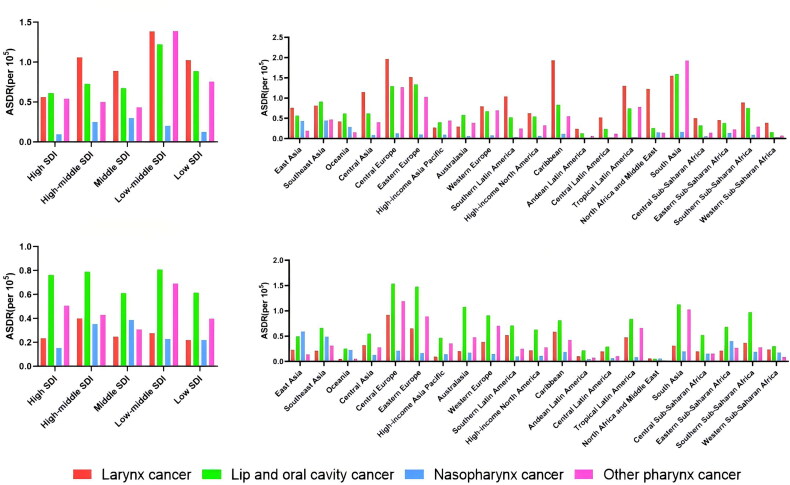
Age-standardized death rate(ASDR) of head and neck cancer by smoking and alcohol use in 21 GBD regions and 5 SDI regions in 2019. (A) Distribution of the effects of smoking on ASDR in head and neck cancer in 5 SDI regions; (B) Distribution of the effects of alcohol use on ASDR in head and neck cancer in 5 SDI regions; (C) Distribution of the effects of smoking on ASDR in head and neck cancer in 21 GBD regions; (D) Distribution of the effects of alcohol use on ASDR in head and neck cancer in 21 GBD regions;

**Figure 6.**
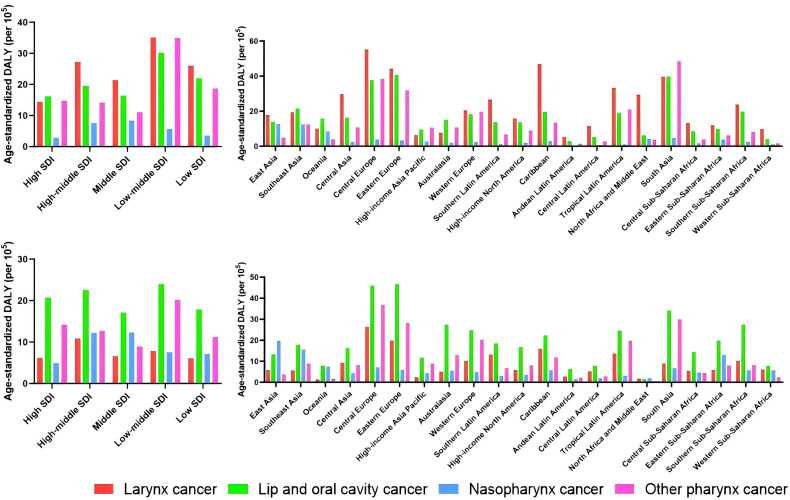
Age-standardized DALY rate of head and neck cancer by smoking and alcohol use in different 21 GBD regions and 5 SDI regions in 2019. (A) Distribution of the effects of smoking on age-standardized DALY rate in head and neck cancer in 5 SDI regions; (B) Distribution of the effects of alcohol use on age-standardized DALY rate in head and neck cancer in 5 SDI regions; (C) Distribution of the effects of smoking on age-standardized DALY rate in head and neck cancer in 21 GBD regions; (D) Distribution of the effects of alcohol use on age-standardized DALY rate in head and neck cancer in 21 GBD regions.

Among the 5 SDI regions, the effect of smoking on ASDR for larynx cancer and lip and oral cavity cancer was higher in the low-middle SDI region than in the other four regions (Figure 5A). The effect of alcohol use on ASDR for larynx cancer was highest in the middle and high SDI regions, whereas the effect on ASDR for lip and oral cavity cancer was highest in the low-middle SDI regions (Figure 5B). The area with middle SDI was the area with the highest influence of smoking and alcohol use on NPC ASDR. It was also the region with the highest age-standardized daly rate for NPC (Figure 6A and B).

Among the 21 GBD regions, the highest ASDR for larynx cancer due to smoking was in Central Europe, followed by the Caribbean (Figure 5C). Larynx cancer ASDR due to alcohol use was also highest in Central Europe, followed by Eastern Europe (Figure 5D). South Asia is the region with the highest ASDR for lip and oral cavity cancers caused by smoking. Central Europe had the highest ASDR for lip and oral cavity cancer due to alcohol use, followed by Eastern Europe. The region with the highest ASDR for NPC caused by alcohol use was East Asia, followed by South Asia (similar to smoking) (Figure 6C and D).

Chewing tobacco is one of important risk factors affecting the disease burden of lip and oral cavity cancer. Among the 5 SDI regions, the low-middle SDI region had the highest ASDR of lip and oral cavity cancer caused by tobacco chewing (Figure S3A). Among the 21 GBD regions, South Asia has the highest age-standardized DALY rate for lip and oral cavity cancer caused by tobacco chewing (Figure S3B).

The proportion of occupational exposure to carcinogens in the disease burden of head and neck cancer is relatively low, but it cannot be ignored. The impact of 0ccupational exposure to asbestos on the disease burden of laryngeal cancer was highest in areas with high SDI. Of the 21 GBD regions, Western Europe (Figure S4A and B). The impact of occupational exposure to sulfuric acid on the disease burden of laryngeal cancer was highest in the low-medddle SDI region compared to the other four SDI regions. Among the 21 GBD regions, the Caribbean had the highest disease burden of laryngeal cancer, followed by South Asia (Figure S5A and B). Occupational exposure to formaldehyde is a carcinogenic risk factor for NPC. The high-medddle region had the highest disease burden of NPC caused by formaldehyde. Among the 21 GBD regions, it was Southeast Asia, followed by East Asia (Figure S6A and B).

## Discussion

The GBD database was created by the Institute for Health Metrics and Evaluation (IHME), an independent population health research organization at the University of Washington School of Medicine, which works with collaborators around the world to develop timely, relevant, and scientifically valid mathematical models that illuminate health everywhere. They inform health policy and practice to achieve their vision of a long and healthy life for all people. (https://www.healthdata.org/about)

Based on the data from the GBD database, this study conducted an in-depth analysis of the risk factors for head and neck malignancies from multiple perspectives including gender, age, and region. We found that smoking and alcohol use were two major risk factors for head and neck cancers. Although the proportion of exposure to carcinogens is relatively small, it is also a risk factor that cannot be ignored.

The harmful substances in tobacco caused by smoking can have direct toxic effects on head and neck tissues such as mouth, throat and larynx after being inhaled into the respiratory tract [8,9]. These harmful substances can cause DNA damage and gene mutations in cells, and eventually lead to the malignant transformation of cells. In addition, smoking increases the risk of head and neck malignancies, especially for long-term, heavy smokers [10,11]. Our study found that smoking was more harmful to men than women in all head and neck cancers, which was related to the greater number of male smokers in the population than women. Studies have shown that men are more likely to be addicted to smoking than women, which further aggravate the risk of smoking on head and neck cancer in men [12,13].

Through data analysis, we found that tobacco chewing was the only risk factor for lip and oral cancers among head and neck cancers. Among women, especially in South Asia, tobacco chewing is the most harmful. We know that the climate and soil conditions in Southeast Asia are very suitable for tobacco cultivation, and tobacco is also an important cash crop in many countries in the region [14]. In some countries, chewing tobacco is a common habit, especially among women [15,16]. Therefore, for women in South Asia, chewing tobacco significantly increases the risk of malignancies such as oral cancer. It is suggested that women should avoid chewing tobacco and cultivate good oral hygiene habits to reduce the risk of disease [17].

Alcohol consumption is another major risk factor for head and neck cancers. Alcohol consumption, especially chronic and heavy drinking, has been scientifically linked to a variety of health problems, including an increased risk of head and neck cancers. First of all, alcohol itself is cytotoxic, which can directly stimulate the mucosa tissues of the mouth and throat. Secondly, alcohol can be a solvent for some carcinogens, and long-term heavy drinking will increase the absorption of these carcinogens. Acetaldehyde, a metabolite of alcohol, is then toxic and capable of damaging DNA and hindering its repair, thereby increasing the likelihood of carcinogenesis [18]. We found that in nasopharyngeal carcinoma, unlike the other two head and neck cancers, alcohol use was nearly consistently slightly more harmful than smoking. The possible reason is that smoking and drinking jointly increase the risk of nasopharyngeal carcinoma through different mechanisms, and their harms strengthen each other [19].

This study analyzed the risk factors of three carcinogens, namely asbestos, sulfuric acid and formaldehyde. Asbestos deposits in tissues and causes damage to cellular structures, leading to cancer risk [20,21]. The carcinogenicity of sulfuric acid mainly lies in the inhalation of inorganic strong acid mist resulting in local low PH may damage DNA and increase the risk of cancer [22]. Formaldehyde can block DNA replication, causing DNA strand breaks and inducing cancer [23,24]. Although the harm degree of these risk factors in head and neck cancer is far less than that of smoking and drinking, they are also risk factors that cannot be ignored.

The limitations of this study are as follows: 1. Thyroid cancer belongs to the category of head and neck cancer, but the risk factors related to thyroid cancer were not discussed in this study. The reason is that high body mass index (BMI) is the only risk factor associated with thyroid cancer in the GBD database, and recent studies have shown that obesity and overweight are associated with an increased risk of thyroid cancer [25]. However, this is not in common with the risk factors discussed in this study for laryngeal cancer, lip and oral cavity cancer, and nasopharyngeal cancer. (2) Human papillomavirus (HPV) infection is a risk factor for laryngeal cancer [26], and Epstein-barr virus (EBV) infection is closely related to the occurrence and development of NPC [11]. However, there is no data on the association between these two types of viruses and laryngeal cancer and NPC in the GBD database.We look forward to the establishment of GBD database for the completion of these aspects of data. In addition, HPV infection is a significant risk factor for head and neck cancers, especially oropharyngeal cancers. Some high-risk types of HPV, such as HPV 16, can integrate into the genome of host cells and interfere with cell growth regulatory genes, leading to uncontrolled cell proliferation and carcinogenesis. Certain genetic predisactions have also been associated with the development of head and neck cancer. For example, people with a family history of head and neck cancer have a relatively high risk of developing the disease, which may be related to inherited genetic variations that make these people more sensitive to carcinogenic factors.

## Conclusion

This study has identified tobacco and alcohol use consumption as the primary risk factors for head and neck cancers, highlighting significant variations in their impact across different cancer species,genders, age groups, and regions. Although the proportion of occupational exposure to some carcinogens is not high, it cannot be ignored. Our findings underscore the critical role of these lifestyle factors in contributing to the incidence, mortality, and overall disease burden of head and neck cancers. The implications of these results are profound, emphasizing the importance of smoking cessation and alcohol moderation as key strategies for the prevention of these cancers. By reducing the prevalence of smoking and excessive alcohol consumption, public health initiatives can effectively lower the incidence and mortality rates of head and neck cancers, thereby alleviating the associated disease burden.

## Supplementary Material

IANN-2024-2627.R1-Fig_S2.jpg

Table S1final.docx

IANN-2024-2627.R1-Fig_S5.jpg

IANN-2024-2627.R1-Fig_S1.jpg

IANN-2024-2627.R1-Fig_S4.jpg

IANN-2024-2627.R1-Fig_S6.jpg

IANN-2024-2627.R1-Fig_S3.jpg

## Data Availability

All the data presented in this manuscript can be downloaded from the official website of the GBD database(https://vizhub.healthdata.org/gbd-results/). Readers can also get the data they want by emailing the corresponding author.

## Statement

Confirm that our research complies with the Declaration of Helsinki. https://www.wma.net/policies-post/wmadeclaration-of-helsinki-ethical-principles-for-medical-research-involving-human-subjects/
