# Supplementary figures and images for: Tobacco and alcohol use are the risk factors responsible for the greatest burden of head and neck cancers: a study from the Global Burden of Disease Study 2019
Source: Ann Med. 2025 May 3;57(1):2500693. doi: 10.1080/07853890.2025.2500693 (PMC12051598; doi:10.1080/07853890.2025.2500693)

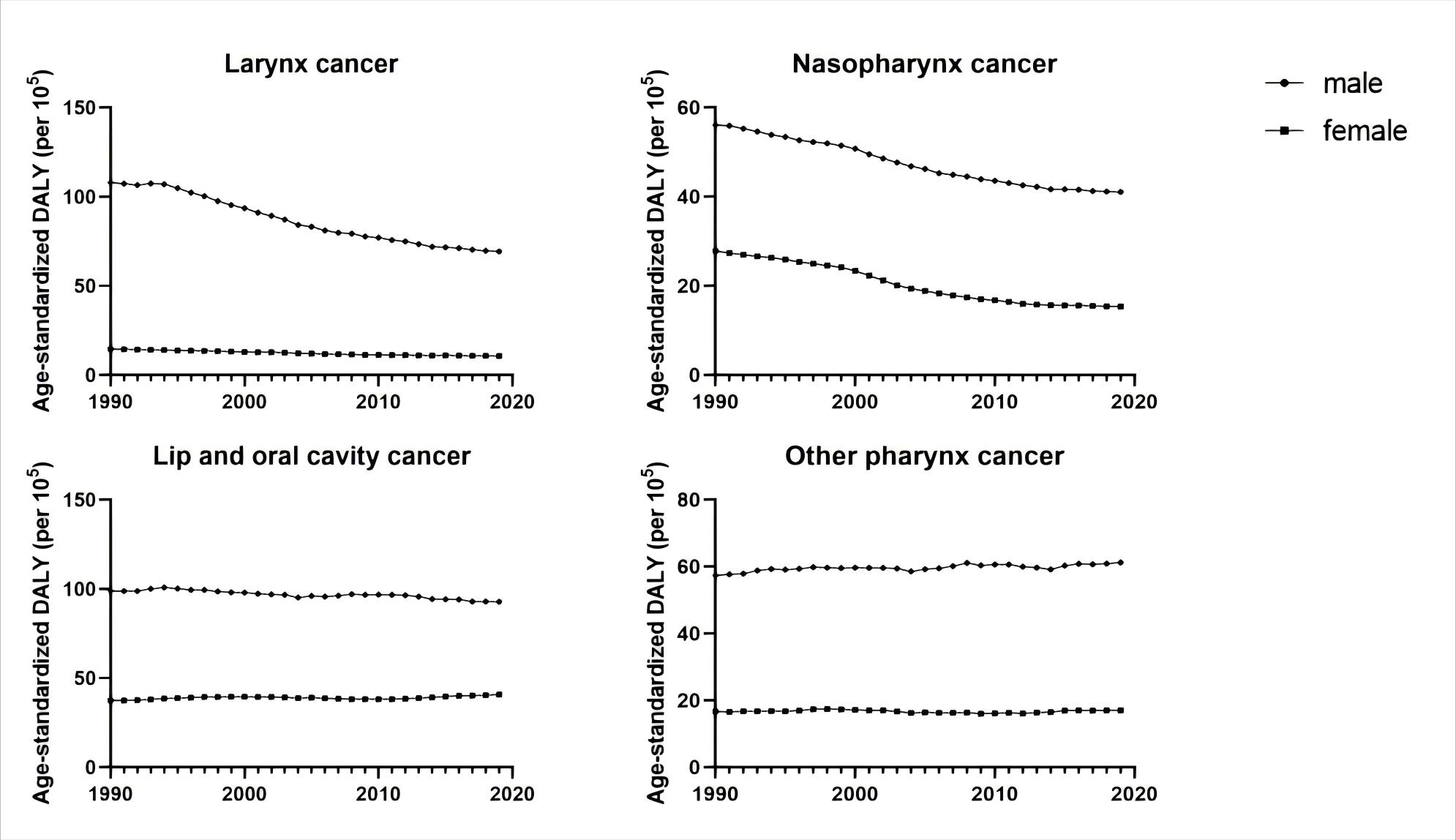

Supplement: IANN-2024-2627.R1-Fig_S2.jpg [file IANN_A_2500693_SM5847.jpg]

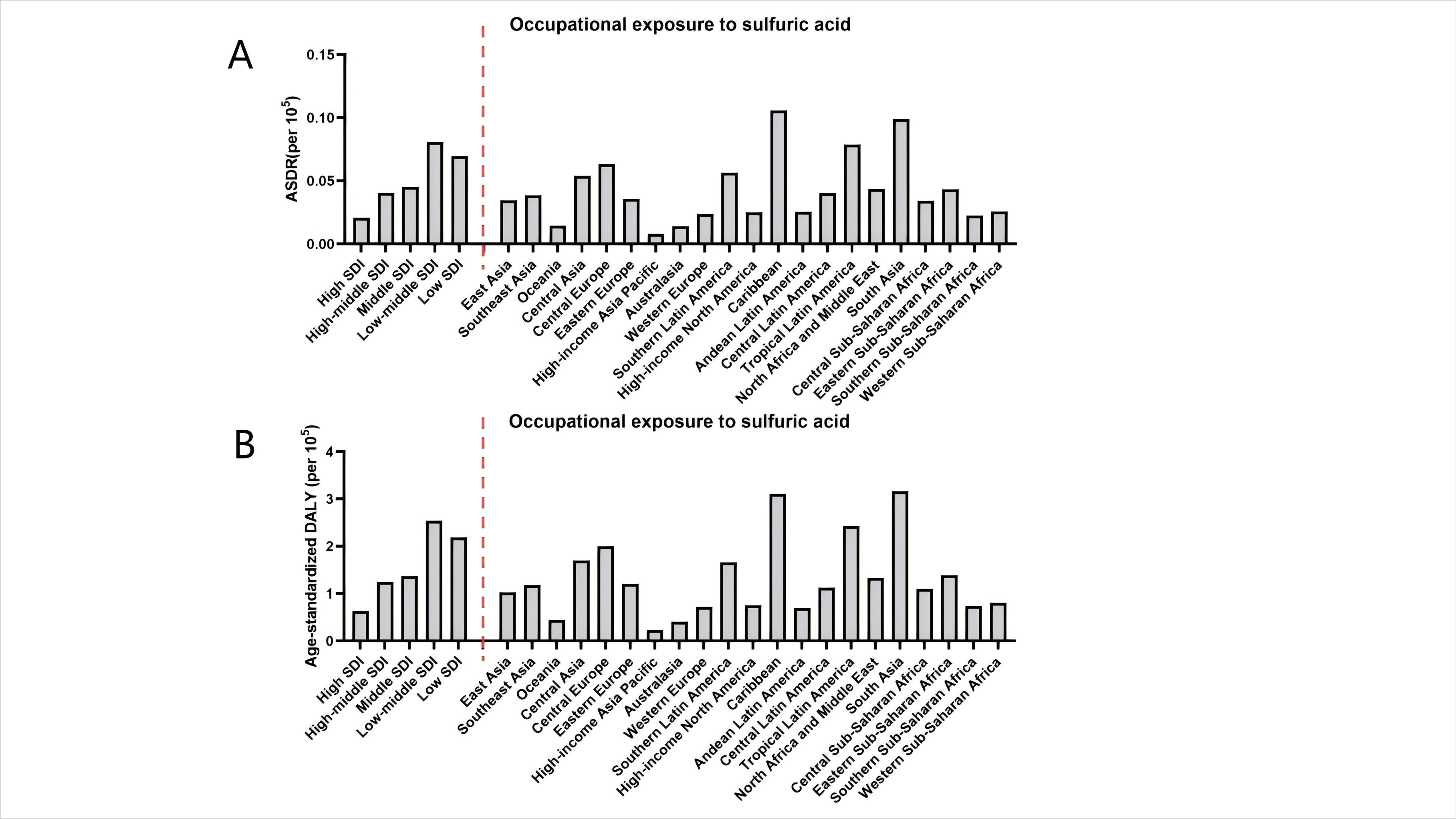

Supplement: IANN-2024-2627.R1-Fig_S5.jpg [file IANN_A_2500693_SM5845.jpg]

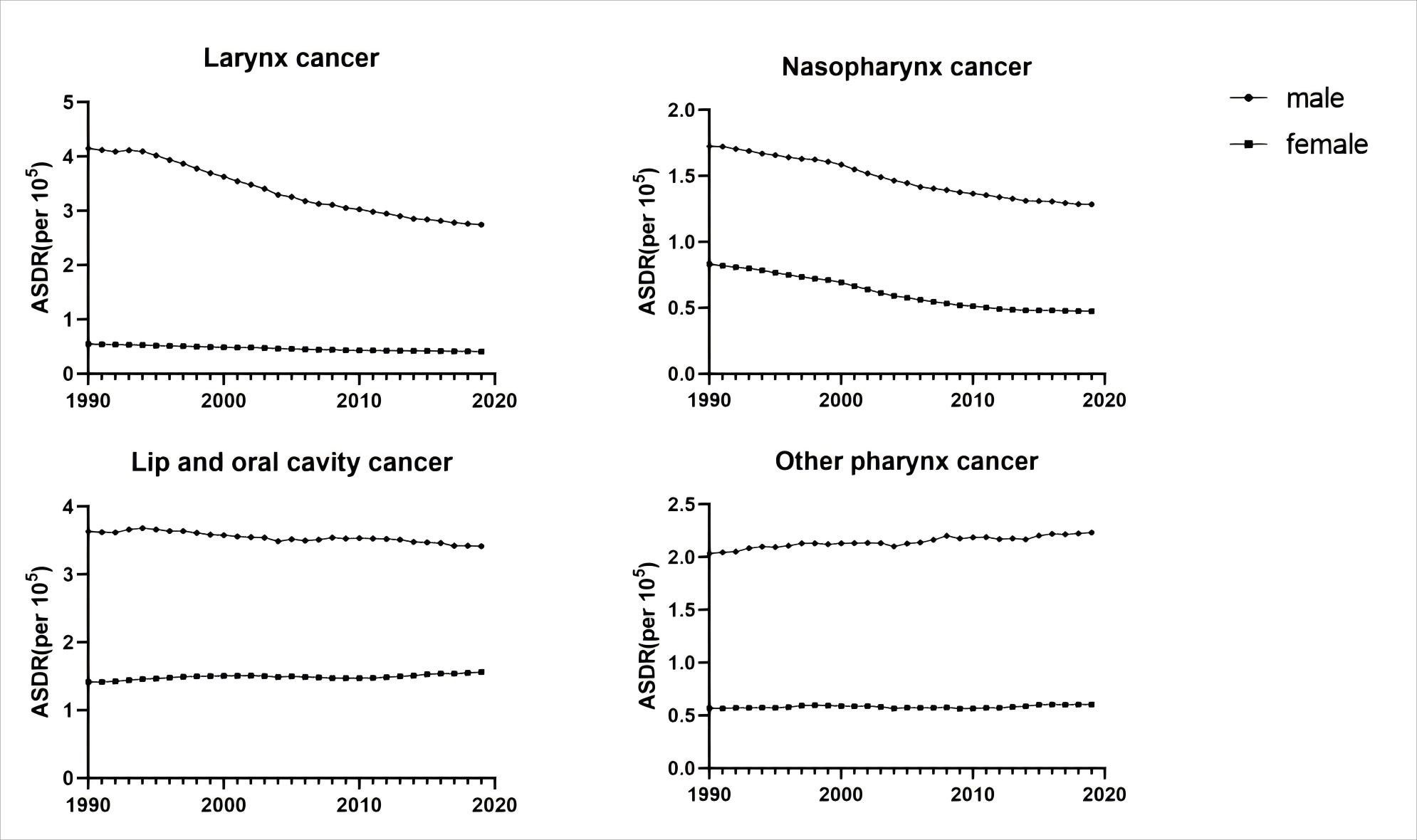

Supplement: IANN-2024-2627.R1-Fig_S1.jpg [file IANN_A_2500693_SM5844.jpg]

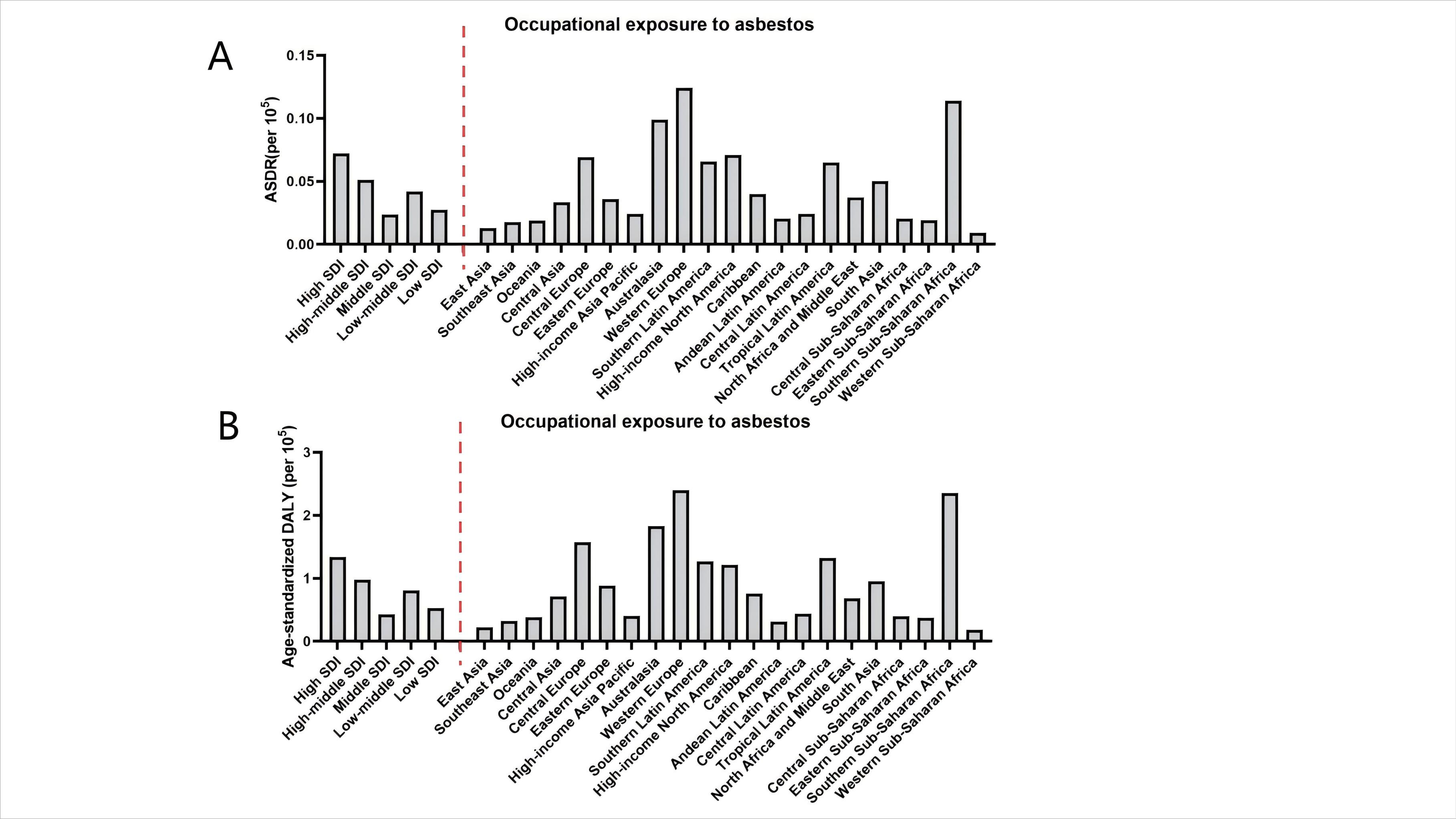

Supplement: IANN-2024-2627.R1-Fig_S4.jpg [file IANN_A_2500693_SM5843.jpg]

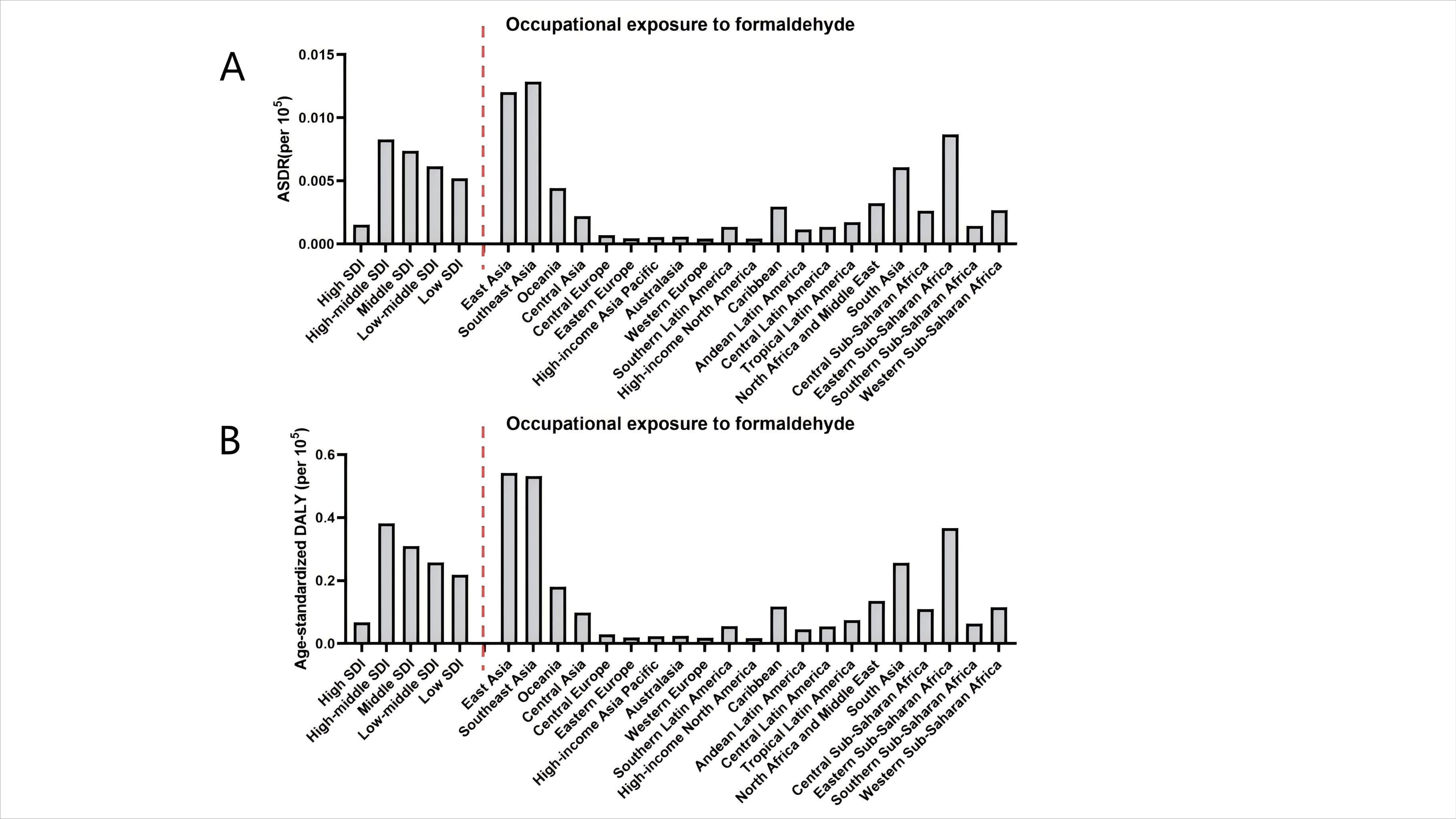

Supplement: IANN-2024-2627.R1-Fig_S6.jpg [file IANN_A_2500693_SM5842.jpg]

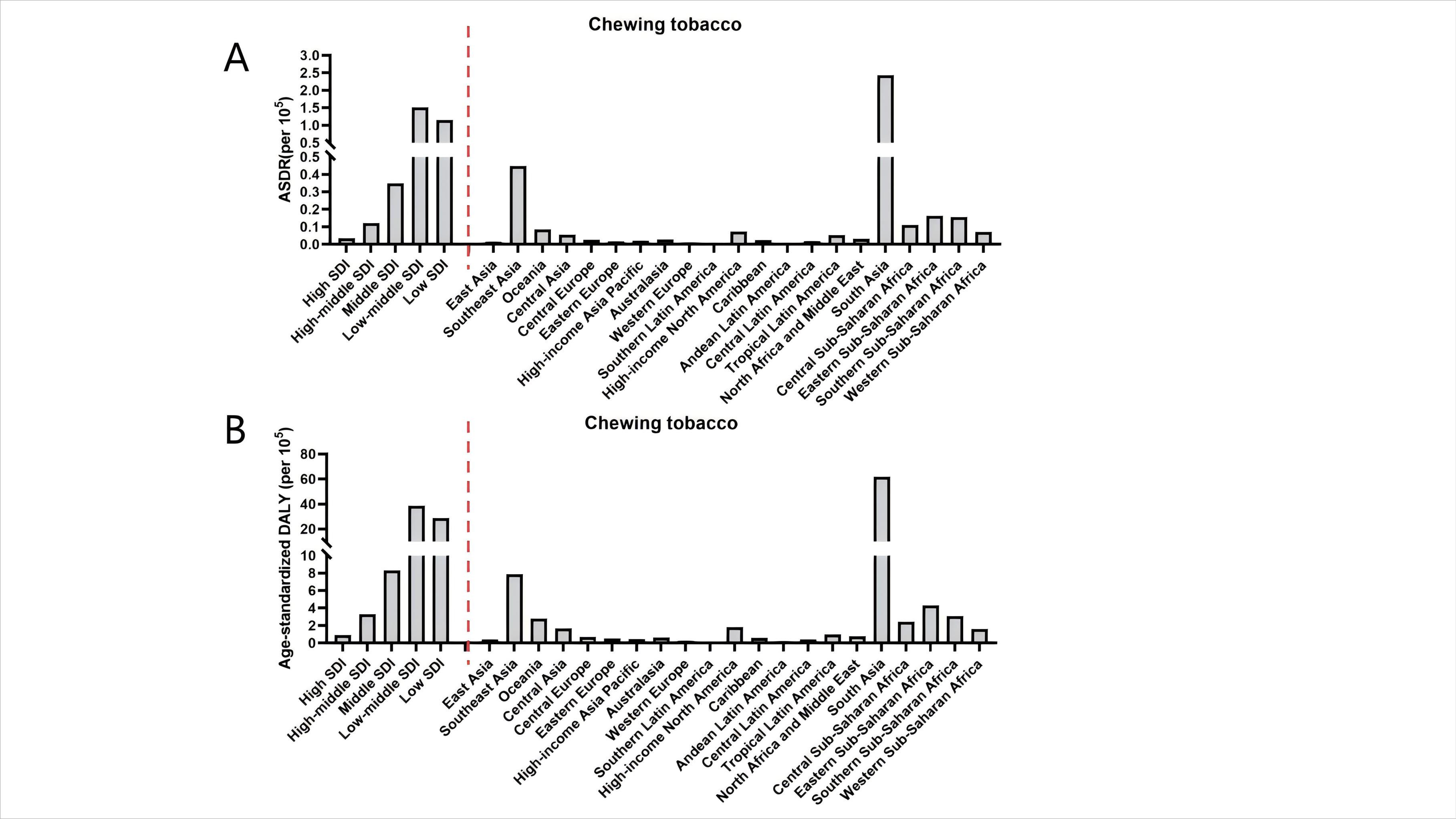

Supplement: IANN-2024-2627.R1-Fig_S3.jpg [file IANN_A_2500693_SM5841.jpg]
