# Supplementary material for: Tobacco and alcohol use are the risk factors responsible for the greatest burden of head and neck cancers: a study from the Global Burden of Disease Study 2019
Source: Ann Med. 2025 May 3;57(1):2500693. doi: 10.1080/07853890.2025.2500693 (PMC12051598; doi:10.1080/07853890.2025.2500693)
Supplement: Table S1final.docx [file IANN_A_2500693_SM5846.docx]

Table S1:The number of global death case and disability-adjusted life years(DALYs) and percent changes for risk factors for head and neck cancer.

| Measure# | Year | Risk factor | Cause(All ages,number) | | | |
| --- | --- | --- | --- | --- | --- | --- |
|  |  |  | Nasopharynx cancer | Lip and oral cavity cancer | Larynx cancer | Other pharynx cancer |
| Deaths | % Change* | Tobacco | 39.46 (19.32 to 64.98) | 91.92 (64.97 to 119.48) | 24.98 (13.99 to 36.61) | 98.36 (67.55 to 126.39) |
|  |  | Smoking | 39.46 (19.32 to 64.98) | 69.57 (45.42 to 93.15) | 24.98 (13.99 to 36.61) | 98.36 (67.55 to 126.39) |
|  |  | Chewing tobacco |  | 141.91 (94.54 to 193.23) |  |  |
|  |  | Alcohol use | 54.18 (29.61 to 82.14) | 104.77 (78.65 to 130.20) | 29.04 (17.04 to 41.93) | 130.67 (163.95 to 98.84) |
|  |  | Occupational carcinogens | 35.22 (13.70 to 64.08) |  | 39.31 (25.41 to 54.44) |  |
|  |  | Occupational exposure to asbestos |  |  | 34.34 (21.14 to 47.37) |  |
|  |  | Occupational exposure to sulfuric acid |  |  | 43.95 (23.90 to 70.48) |  |
|  |  | Occupational exposure to formaldehyde | 35.22 (13.70 to 64.08) |  |  |  |
|  | 2019 | Tobacco | 17917.96 (12960.97 to 23029.24) | 92660.28 (78829.68 to 107043.64) | 78269.08 (67968.78 to 88323.69) | 53612.44 (45182.27 to 61925.36) |
|  |  | Smoking | 17917.96 (12960.97 to 23029.24) | 63433.56 (51216.43 to 76386.46) | 78269.08 (67968.78 to 88323.69) | 53612.44 (45182.27 to 61925.36) |
|  |  | Chewing tobacco |  | 37315.20 (27891.90 to 47220.70) |  |  |
|  |  | Alcohol use | 24458.84 (18824.16 to 29947.73) | 60363.57 (47971.76 to 72430.53) | 23892.67 (14144.18 to 32627.43) | 37867.89 (29032.87 to 46970.33) |
|  |  | Occupational carcinogens | 518.35 (354.81 to 731.08) |  | 7645.91 (4692.16 to 11406.39) |  |
|  |  | Occupational exposure to asbestos |  |  | 3682.09 (2035.39 to 5527.70) |  |
|  |  | Occupational exposure to sulfuric acid |  |  | 4032.29 (1730.16 to 7465.23) |  |
|  |  | Occupational exposure to formaldehyde | 518.35 (354.81 to 731.08) |  |  |  |
| DALYs | % Change | Tobacco | 33.66 (13.63 to 59.02) | 80.73 (54.03 to 107.88) | 16.23 (5.81 to 107.88) | 85.00 (56.59 to 112.12) |
|  |  | Smoking | 33.66 (13.63 to 59.02) | 59.16 (35.88 to 82.20) | 16.23 (5.81 to 107.88) | 85.00 (56.59 to 112.12) |
|  |  | Chewing tobacco |  | 126.78 (81.14 to 176.36) |  |  |
|  |  | Alcohol use | 47.30 (24.10 to 74.29) | 93.76 (67.66 to 119.53) | 21.13 (9.76 to 33.65) | 118.20 (88.14 to 150.84) |
|  |  | Occupational carcinogens | 30.55 (9.69 to 58.18) |  | 31.75 (16.80 to 47.28) |  |
|  |  | Occupational exposure to asbestos |  |  | 20.88 (8.37 to 33.54) |  |
|  |  | Occupational exposure to sulfuric acid |  |  | 38.48 (18.55 to 64.66) |  |
|  |  | Occupational exposure to formaldehyde | 30.55 (9.69 to 58.18) |  |  |  |
|  | 2019 | Tobacco | 526562.79 (374036.93 to 684218.45) | 2472249.76 (2082247.39 to 2897800.74) | 2023840.72 (1756894.60 to 2296066.72) | 1442985.60 (1200428.29 to 1684490.56) |
|  |  | Smoking | 526562.79 (374036.93 to 684218.45) | 1656259.97 (1310830.93 to 2015843.95) | 2023840.72 (1756894.60 to 2296066.72) | 1442985.60 (1200428.29 to 1684490.56) |
|  |  | Chewing tobacco |  | 1028540.70 (763640.58 to 1318941.57) |  |  |
|  |  | Alcohol use | 810543.63 (627787.06 to 993042.30) | 1726579.57 (1376900.89 to 2066136.75) | 655857.83 (395281.34 to 894735.41) | 1106508.31 (853580.62 to 1367005.43) |
|  |  | Occupational carcinogens | 22336.62 (15068.55 to 31498.79) |  | 194607.22 (114854.93 to 306133.84) |  |
|  |  | Occupational exposure to asbestos |  |  | 70003.67 (38275.87 to 106450.25) |  |
|  |  | Occupational exposure to sulfuric acid |  |  | 126341.40 (54057.77 to 233589.14) |  |
|  |  | Occupational exposure to formaldehyde | 22336.62 (15068.55 to 31498.79) |  |  |  |

#Data in parentheses are 95% Uncertainty Intervals (95% UIs).

*% Change(1990–2019).
